# A complicated prosthetic valve endocarditis due to methicillin resistant *Staphylococci* treated with linezolid and ciprofloxacin: a case report

**DOI:** 10.1186/s13104-017-2907-z

**Published:** 2017-11-09

**Authors:** C. G. K. Amiyangoda, H. Wimalaratna, S. Bowatte

**Affiliations:** Teaching Hospital, Kandy, Sri Lanka

**Keywords:** Prosthetic valve endocarditis, Methicillin resistant *staphylococci*, Linezolid, Sri Lanka

## Abstract

**Background:**

Prosthetic valve endocarditis (PVE) due to methicillin resistant *Staphylococcus aureus* (MRSA) is a rare disease with significant mortality and morbidity. With the emerging resistance and adverse effect profile of vancomycin which is the standard treatment, there is a compelling necessity of an effective alternative for vancomycin. Linezolid is proved as such an agent for infections caused by MRSA in other sites. However to-date the evidence for successful use of linezolid for MRSA prosthetic valve endocarditis is limited only for few case studies. We here present the third case reported as effective treatment of PVE by MRSA with linezolid and probably the first case reported with successful treatment with linezolid in a patient with multiple complications who is a candidate for surgery in standard guidelines.

**Case presentation:**

A 45 years old male from Kandy Sri Lanka, who had undergone prosthetic valve replacement 10 years back, presented with prosthetic mitral valve endocarditis caused by MRSA. He failed to respond to vancomycin and cotrimoxazole while sustaining cerebral haemorrhages, as well as life threatening ventricular arrhythmias. Treatment with intravenous linezolid and ciprofloxacin resulted in a complete response with disappearance of the vegetations and sterilization of blood cultures.

**Conclusions:**

Linezolid can be considered as a good option for treating PVE by MRSA infections who are not responding to vancomycin and may negate the need for a surgery in patients awaiting an early surgery. Further studies including randomized controlled trials are needed to assess the efficacy of linezolid in PVE due to MRSA.

## Background

Prosthetic valve endocarditis (PVE) is considered as a relatively rare disorder with significant mortality and morbidity [1–3]. Management of PVE becomes more challenging when it is caused by methicillin resistant *Staphylococcus aureus* (MRSA) particularly due to the associated worse clinical outcome as well as the limited availability of therapeutic options [4, 5]. Currently the first line treatment for PVE-IE caused by MRSA includes the combination of vancomycin, rifampicin and aminoglycosides. If the patient is not responding to medical treatment or complications develop, early surgery should be considered [5]. Successful treatment of PVE with newer antibiotics following a failure in responsiveness to first line therapy has been reported [6, 7]. Of these only few cases of MRSA-PVE are reported as successful treatment with alternative antibiotics [8, 9]. In this report we describe a patient, with a prosthetic mitral valve presenting with infective endocarditis (IE) caused by MRSA complicated by intracranial haemorrhages and frequent ventricular arrhythmias, who was successfully treated with linezolid without requiring a valve replacement.

## Case presentation

In April 2015 a 45 years old male who had undergone a metallic prosthetic valve replacement 10 years ago presented to a medical unit at Teaching Hospital, Kandy, Sri Lanka with fever for 5 days duration. There was no history of intravenous drug abuse. He had a diastolic murmur at the apex with muffled metallic sound of prosthetic valves and examination of the optic fundi revealed a Roth’s spot in the right eye. Investigations showed a leukocyte count of 10,000/µl with thrombocytopenia (platelet count of 117,000/µl) and elevated C reactive protein (CRP). Urine analysis showed red cells with red cell casts and urine culture was negative. Blood cultures yielded *Staphylococci* which were resistant to methicillin and sensitive to vancomycin with an MIC of 0.25 µg/ml. Transthoracic echocardiogram showed two mobile vegetations (10.3 × 3.7 mm and 12.4 × 1.3 mm in size) attached to prosthetic mitral valve and further a transoesophageal echocardiogram confirmed these findings. He was started on intravenous vancomycin, gentamicin and oral rifampicin.

**Fig. 1:**
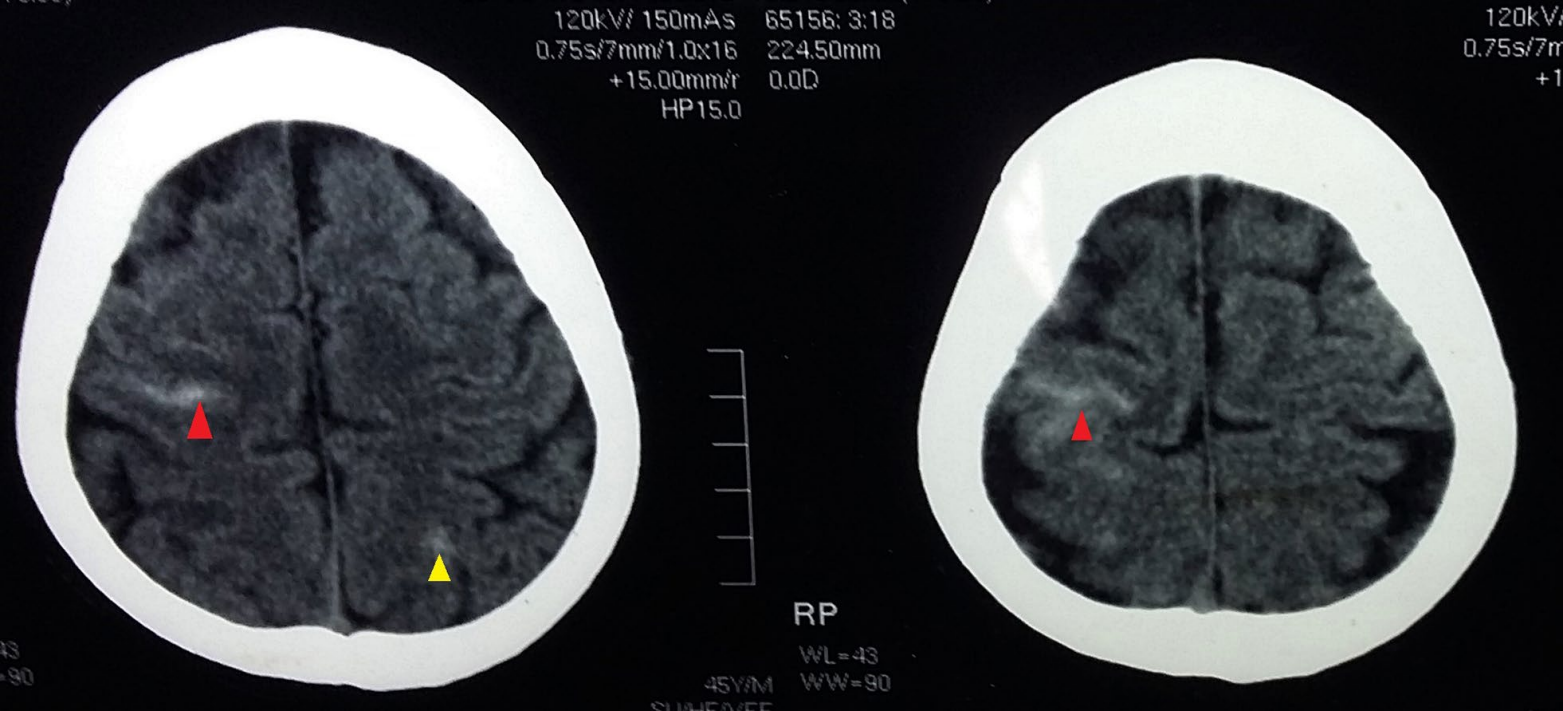
Computed tomography (CT) scan of the head: showing subarachnoid (red) and intracranial haemorrhages (yellow)

Despite the intravenous antibiotics with adequate vancomycin trough serum levels he remained bacteremic with continuing fever and persistent isolation of MRSA in blood cultures, and high levels of CRP. On the fifth day of admission, patient complained of severe headache and computed tomography (CT) scan of head revealed a subarachnoid haemorrhage and a intracranial haemorrhage possibly following rupture of mycotic aneurysms (Fig. 1). At that moment patient was considered for surgical treatment as he had persistent bacteraemia along with multiple embolic events. However, considering the suggestions of the multi-disciplinary team (microbiologist, neurosurgeon and cardiothoracic surgeon) the decision was made to continue the total course of antibiotics while planning for an early surgery since there was no urgent or emergency indication for a surgery. A second surgery at this point would have been more difficult and operating on an infected field carries a high risk of postoperative complications. Moreover since the patient was having intracranial haemorrhage he was at a significant risk of mortality and adverse outcome during as well as after an early surgery. In addition to the previous antibiotics he was started on oral co-trimoxazole as well. On day 15 of hospitalization there was a failure in settling the fever and resolution of bacteremia.

Fifteen days after admission, his antibiotic regimen was modified with linezolid/cipro initiation while all other antibiotics were stopped since there was no response to the treatment. He was started on intravenous linezolid 600 mg 8 hourly. Intravenous ciprofloxacin was also added to linezolid. Subsequently the patient developed frequent ventricular ectopics and Holter study revealed a heavy burden of ventricular ectopics including short runs of nonsustained ventricular tachycardia. With the opinion of the consultant electrophysiologist, he was loaded with intravenous amiodarone for which there was a reduction in the ectopic burden and he was then maintained on oral amiodarone. By this time a gradual reduction of the height of the fever spikes was noticed indicating a response to the new course of antibiotics, and after 7 days of linezolid treatment the temperature became normal and CRP level improved. Blood culture became negative on day 9 of linezolid treatment. He was continued on intravenous linezolid and ciprofloxacin for a total of 4 weeks and then converted to oral linezolid and ciprofloxacin to complete the total duration of antibiotics of 6 weeks. The patient didn’t develop any significant adverse drug reaction to the treatment. The follow up transthoracic echocardiogram revealed no vegetations and to-date the patient is being regularly followed up at our clinic with no further complications.

## Discussion

Prosthetic valve endocarditis is a rare disease with a significant mortality and morbidity in spite of improvement in the diagnosis, prophylaxis and treatment over time [3]. When the prosthetic valves are infected with methicillin resistant *staphylococci* it encompasses a significant challenge to the treating clinician for the debate on most appropriate and effective management for the latter is still ongoing. Traditionally vancomycin has been the recommended antibiotic for MRSA-PVE along with gentamicin and rifampicin [4, 5]. However the recent emergence of vancomycin resistance and its adverse effect profile have lead to the introduction of newer antibiotics against MRSA including linezolid, daptomycin and cotrimoxazole. A recent meta-analysis of randomized controlled trials has shown that linezolid has a significant advantage over vancomycin with respect to drug efficacy and could possibly be considered as a superior alternative for MRSA infections [10]. However the study does not contain patients with infective endocarditis and there are no other randomized controlled trials available on treatment of IE or PVE caused by MRSA thus far.

Linezolid is a synthetic oxazolidinone which has a broad spectrum of activity against gram positive bacteria and has less adverse effects compared to vancomycin. Linezolid inhibits protein synthesis with an oral bioavailability of 100%. Interestingly it has shown only limited emergence of drug resistance which would be a major therapeutic advantage. There is limited evidence in the literature regarding the use of linezolid in IE by MRSA. In an experimental rabbit model of native valve infective endocarditis which compared the efficacy of vancomycin alone, linezolid alone and combination of linezolid and vancomycin has shown a higher efficacy of treatment with vancomycin alone [11]. Another systemic review was done to assess the outcome of treatment of infective endocarditis with linezolid in 2006 described only one patient with PVE due to MRSA who was successfully treated with linezolid without the need for valve replacement [12]. In a review done by Howden et al. has assessed the use of linezolid in eight patients with infective endocarditis and successful treatment has been shown only in four patients who had native valve endocarditis and in one patient vegetectomy was done. The review contained only one patient with prosthetic valve endocarditis who was treated with linezolid which ultimately resulted in redo aortic valve replacement and unfortunately the patient had died of post operative bleeding [13]. Linezolid failure has been reported in another two patients with native valve endocarditis [14].

Valve replacement in PVE IE is a matter of considerable debate. According to the current guidelines, our patient had enough recommended indications for surgery; he was not responding to the effective doses of susceptible antibiotics for more than 7 days with vegetations of more than 1 cm in size and mobile in nature, and having multiple cerebral emboli [5]. In a propensity analysis of a multicenter, international cohort study to assess the use and effect of surgical therapy for prosthetic valve infective endocarditis has shown that the in hospital mortality was mainly predicted by brain embolization and *Staphylococcus aureus* infection with a shown benefit by surgery. But the limitation of the study was that the timing of the surgical intervention was not included in the analysis and also the data regarding the antibiotics used prior to surgery were not compared with the patients who needed surgical treatment [15]. Even though the currently available guidelines may help clinicians in deciding whether and when patients with IE should undergo surgery, such decisions can be extremely difficult in individual and unique patients, and particularly in patients with PVE. In a prospective cohort study it has shown that early valve surgery is not an independent predictor of reduced mortality in patients with staphylococcus PVE. The decisions about valve surgery in patients with staphylococcus PVE should be individualized for each patient and be based on a careful clinical multidisciplinary evaluation [16].

In summary only a limited number of cases have been reported regarding the successful treatment of MRSA-PVE using linezolid thus far, and the current report highlights the successful medical treatment of a patient having multiple complications with considerable indications for surgery. To our knowledge this is the third case report on successful treatment of MRSA-PVE with linezolid and is the only report describing survival of a patient with medical treatment in spite of multiple complications warranting for surgery.

## Conclusions

There are only limited evidence on effective alternatives for treatment of MRSA-PVE. Our case illustrates that linezolid can be considered as an option for vancomycin resistance in treating patients with MRSA-PVE and may negate the need for a valve surgery. These evidence warrant further extensive investigation on this therapeutic approach towards a successful combat against MRSA-PVE.

## Abbreviations

CRP: reactive protein
IE: infective endocarditis
MRSA: methicillin resistant *Staphylococcus aureus*
PVE: prosthetic valve endocarditis
WBC: white blood cell

## References

[1] Tornos P, Almirante B, Olona M, Permanyer G, González T, Carballo J, Pahissa A, Soler-Soler J (1997). Clinical outcome and long-term prognosis of late prosthetic valve endocarditis: a 20-year experience. Clin Infect Dis.

[2] Sy R, Chawantanpipat C, Richmond D, Kritharides L (2011). Development and validation of a time-dependent risk model for predicting mortality in infective endocarditis. Eur Heart J.

[3] Alonso-Valle H, Fariñas-Álvarez C, García-Palomo JD, Bernal JM, Martín-Durán R, Díez JF, Revuelta JM, Fariñas MC (2010). Clinical course and predictors of death in prosthetic valve endocarditis over a 20-year period. J Thorac Cardiovasc Surg.

[4] Boucher HW, Corey GR (2008). Epidemiology of methicillin-resistant *Staphylococcus aureus*. Clin Infect Dis.

[5] Baddour LM, Wilson WR, Bayer AS, Fowler VG, Tleyjeh IM, Rybak MJ, Barsic B, Lockhart PB, Gewitz MH, Levison ME, Bolger AF (2015). Infective endocarditis in adults: diagnosis, antimicrobial therapy, and management of complications. Circulation.

[6] Souli M, Pontikis K, Chryssouli Z, Galani I, Giamarellou H (2005). Successful treatment of right-sided prosthetic valve endocarditis due to methicillin-resistant teicoplanin-heteroresistant *Staphylococcus aureus* with linezolid. Eur J Clin Microbiol Infect Dis.

[7] Leung KT, Tong MK, Siu YP, Lam CS, Ng HL, Lee HK (2004). Treatment of vancomycin-intermediate *Staphylococcus aureus* endocarditis with linezolid. Scand J Infect Dis.

[8] Cheung LL, Yue CS, Fung K, Chu CM, Tso EY (2011). Daptomycin as successful treatment for a refractory case of prosthetic valve endocarditis because of methicillin-sensitive *Staphylococcus aureus*. Heart Lung J Acute Crit Care.

[9] Mohan SS, McDermott BP, Cunha BA (2005). Methicillin-resistant *Staphylococcus aureus* prosthetic aortic valve endocarditis with paravalvular abscess treated with daptomycin. Heart Lung J Acute Crit Care.

[10] An MM, Shen H, Zhang JD, Xu GT, Jiang YY (2013). Linezolid versus vancomycin for meticillin- resistant *Staphylococcus aureus* infection: a meta-analysis of randomised controlled trials. Int J Antimicrob Agents.

[11] Chiang FY, Climo M (2003). Efficacy of linezolid alone or in combination with vancomycin for treatment of experimental endocarditis due to methicillin-resistant *Staphylococcus aureus*. Antimicrob Agents Chemother.

[12] Falagas ME, Manta KG, Ntziora F, Vardakas KZ (2006). Linezolid for the treatment of patients with endocarditis: a systematic review of the published evidence. J Antimicrob Chemother.

[13] Howden BP, Ward PB, Charles PG, Korman TM, Fuller A, du Cros P, Grabsch EA, Roberts SA, Robson J, Read K, Bak N (2004). Treatment outcomes for serious infections caused by methicillin-resistant *Staphylococcus aureus* with reduced vancomycin susceptibility. Clin Infect Dis.

[14] Ruiz ME, Guerrero IC, Tuazon CU (2002). Endocarditis caused by methicillin-resistant *Staphylococcus aureus*: treatment failure with linezolid. Clin Infect Dis.

[15] Wang A, Pappas P, Anstrom KJ, Abrutyn E, Fowler VG, Hoen B, Miro JM, Corey GR, Olaison L, Stafford JA, Mestres CA (2005). The use and effect of surgical therapy for prosthetic valve infective endocarditis: a propensity analysis of a multicenter, international cohort. Am Heart J.

[16] Chirouze C, Alla F, Fowler VG, Sexton DJ, Corey GR, Chu VH, Wang A, Erpelding ML, Durante-Mangoni E, Fernández-Hidalgo N, Giannitsioti E (2015). Impact of early valve surgery on outcome of *Staphylococcus aureus* prosthetic valve infective endocarditis: analysis in the international collaboration of endocarditis–prospective cohort Study. Clin Infect Dis.

